# Genomic characterization of *Klebsiella* spp. from bovine mastitis: dissemination of a conserved, highly transmissible *lac*_acq_^+^ *fec*^+^ plasmid drives burden of disease

**DOI:** 10.1128/aem.01162-25

**Published:** 2025-11-20

**Authors:** Michael Biggel, Magdalena Nüesch-Inderbinen, Mitsuko Logean, Sabrina Corti, Lucien Kelbert, Roger Stephan

**Affiliations:** 1 Vetsuisse Faculty, Institute for Food Safety and Hygiene, University of Zurich 600627, Zurich, Switzerland; Universidad de los Andes, Bogotá, Colombia

**Keywords:** virulence, *lac* operon, mobile genetic elements, *Klebsiella pneumoniae*, bovine mastitis

## Abstract

Bovine mastitis poses a significant health concern for dairy cattle and a major economic burden on the dairy industry. *Klebsiella* spp. are important mastitis pathogens, with previous studies identifying lactose-utilization (*lac*_acq_) and iron-acquisition (*fec*) systems as key determinants associated with pathogenicity. To investigate how these are acquired and shared, we genomically characterized 60 mastitis-associated *Klebsiella* isolates by fully resolving their chromosomes and plasmids. The isolates were identified as *Klebsiella pneumoniae* (*n* = 46), *Klebsiella michiganensis* (*n* = 8), *Klebsiella quasipneumoniae* (*n* = 3), *Klebsiella grimontii* (*n* = 2), and *Klebsiella variicola* (*n* = 1). Phylogenetic analysis revealed diverse lineages, with sporadic transmission events within and across farms. Among unique *Klebsiella* spp. isolates, 48/55 (87.3%) and 53/55 (96.4%) harbored *lac*_acq_ and *fec*, respectively. In *K. pneumoniae*, the *lac*_acq_ operon was consistently found on plasmids alongside *fec*. Both horizontal and vertical transfer of *lac*_acq_^+^ *fec*^+^ plasmids were observed. Many phylogenetically diverse mastitis isolates from distant farms carried an identical conjugative 132 kb plasmid, suggesting recent acquisitions of a single circulating plasmid as a major driver of mastitis. *K. pneumoniae* ST107, a globally prevalent mastitis-causing lineage, carried large non-mobilizable plasmids with *fec* and two *lac*_acq_ operons. In contrast to *K. pneumoniae*, mastitis-associated *K. michiganensis* often carried *lac*_acq_ on integrative conjugative elements and inherently harbored a chromosomal *fec*-like gene cluster. Few isolates possessed antimicrobial resistance genes or virulence factors linked to pathogenicity in humans. Our results provide new insights into the genomic diversity of mastitis-associated *Klebsiella* and the role of mobile genetic elements.

**IMPORTANCE:**

Understanding the genetic basis of *Klebsiella*-induced mastitis is essential for improving prevention and control strategies. Our study reveals that the key mastitis-associated traits—lactose utilization (*lac*_acq_) and iron acquisition (*fec*)—are commonly encoded on plasmids. The discovery of an identical conjugative plasmid in diverse *Klebsiella pneumoniae* lineages highlights the potential for rapid and widespread dissemination of virulence traits, independent of clonal background. However, we also show that clonal spread—combined with the vertical inheritance of a *lac*_acq_⁺ *fec*⁺ plasmid—contributes to the success of *K. pneumoniae* ST107, a globally prevalent mastitis lineage. Together, our findings highlight the central role of mobile genetic elements in the ecology of mastitis-associated *Klebsiella*.

## INTRODUCTION

*Klebsiella* spp. are important etiological agents of mastitis in dairy cows (1). *Klebsiella pneumoniae*-induced mastitis can present with a rapid onset and an acute inflammatory response with severe clinical signs (2, 3). The efficacy of antimicrobial treatment is often limited by the severity of the disease, often resulting in chronic infections and high mortality rates (3, 4).

In addition to the considerable negative impact on animal health and welfare, bovine mastitis causes high economic losses to the dairy industry due to reduced milk yield and product quality, expenditures for treatment, and the risk of premature culling (5). Globally, subclinical and clinical mastitis result in mean annual losses of approximately 9 billion US dollars and 13 billion US dollars, respectively (6). While economic costs vary between animals and pathogens, production losses are greatest in multiparous cows affected by mastitis caused by *Klebsiella* spp., making these pathogens a particular concern for the dairy industry (3, 7, 8).

Unlike the classic contagious bacteria, such as *Streptococcus agalactiae* and *Staphylococcus aureus*, *Klebsiella* spp. are considered environmental pathogens acquired through exposure to contaminated bedding, drinking water, cow manure, and other contaminated areas (3, 9). Moreover, *Klebsiella* spp. and other opportunistically pathogenic bacteria associated with environmental bovine mastitis are carried by *Stomoxys* (stable flies), which contribute to disease transmission within the dairy barn setting (10).

Virulence factors play a crucial role in the pathogenesis of *Klebsiella* (9, 11, 12). Determinants that are often identified in the accessory genome of human pathogenic *Klebsiella* comprise genes encoding the siderophores aerobactin (*iuc*), yersiniabactin (*ybt*), salmochelin (*iro*), the genotoxin colibactin (*clb*), and the *rmpA*/*rmpA2* locus responsible for the hypermucoid phenotype, among others (13). By contrast, bovine mastitis-associated *Klebsiella* appears for the most part to lack virulence factors associated with severe infection in humans (12, 14). Nevertheless, previous studies have identified two genomic determinants that distinguish bovine from human *Klebsiella*: a ferric citrate iron uptake system (*fecABCDE*) and a lactose utilization operon (here referred to as *lac*_acq_), which some strains acquired in addition to their intrinsic *Klebsiella lac* operon (12, 14, 15). These genetic differences likely reflect adaptation to the host mammary gland, such as the ability to efficiently metabolize lactose through the use of the *lac*_acq_ operon or to use *fec* to sequester iron from milk in competition with the iron-binding lactoferrin, which is produced by the mammary gland during infection (12, 14, 16). To investigate how these determinants are acquired and shared, we collected and sequenced 60 *Klebsiella* strains from distinct bovine mastitis cases from 49 different dairy farms in Switzerland. Short-read and long-read sequencing were used to fully resolve the *Klebsiella* chromosomes and plasmids and to determine species distribution, virulence factors, and antimicrobial resistance genes.

## RESULTS

### Epidemiology and phylogenetic diversity

We sequenced genomes of 60 *Klebsiella* spp. isolates recovered from the milk of 60 cows with acute (*n* = 59) or subclinical (*n* = 1) mastitis, collected across 49 farms in Switzerland from December 2022 to August 2024. Forty-three farms provided a single isolate each, five farms provided two isolates, and one farm (farm A) provided seven isolates. *K. pneumoniae* was the dominant species, accounting for 76.7% (46/60) of the isolates. The remaining isolates belonged to *Klebsiella grimontii* (*n* = 2), *Klebsiella michiganensis* (*n* = 8), *Klebsiella quasipneumoniae* (*n* = 3), and *Klebsiella variicola* (*n* = 1). Core genome multilocus sequence typing (cgMLST) and confirmatory single-nucleotide polymorphism (SNP) analyses indicated sporadic transmission events of *K. pneumoniae* strains: clusters (pairwise distance ≤10 cgMLST alleles and ≤10 cgSNPs) within ST661 (two isolates) and ST299 (three isolates) suggested within-farm transmissions between cows. Both clusters were found at farm A. A third cluster (ST432, three isolates) suggested both within- and across-farm transmissions (Table S1). Five farms yielded multiple but genetically unrelated isolates.

**Fig 1:**
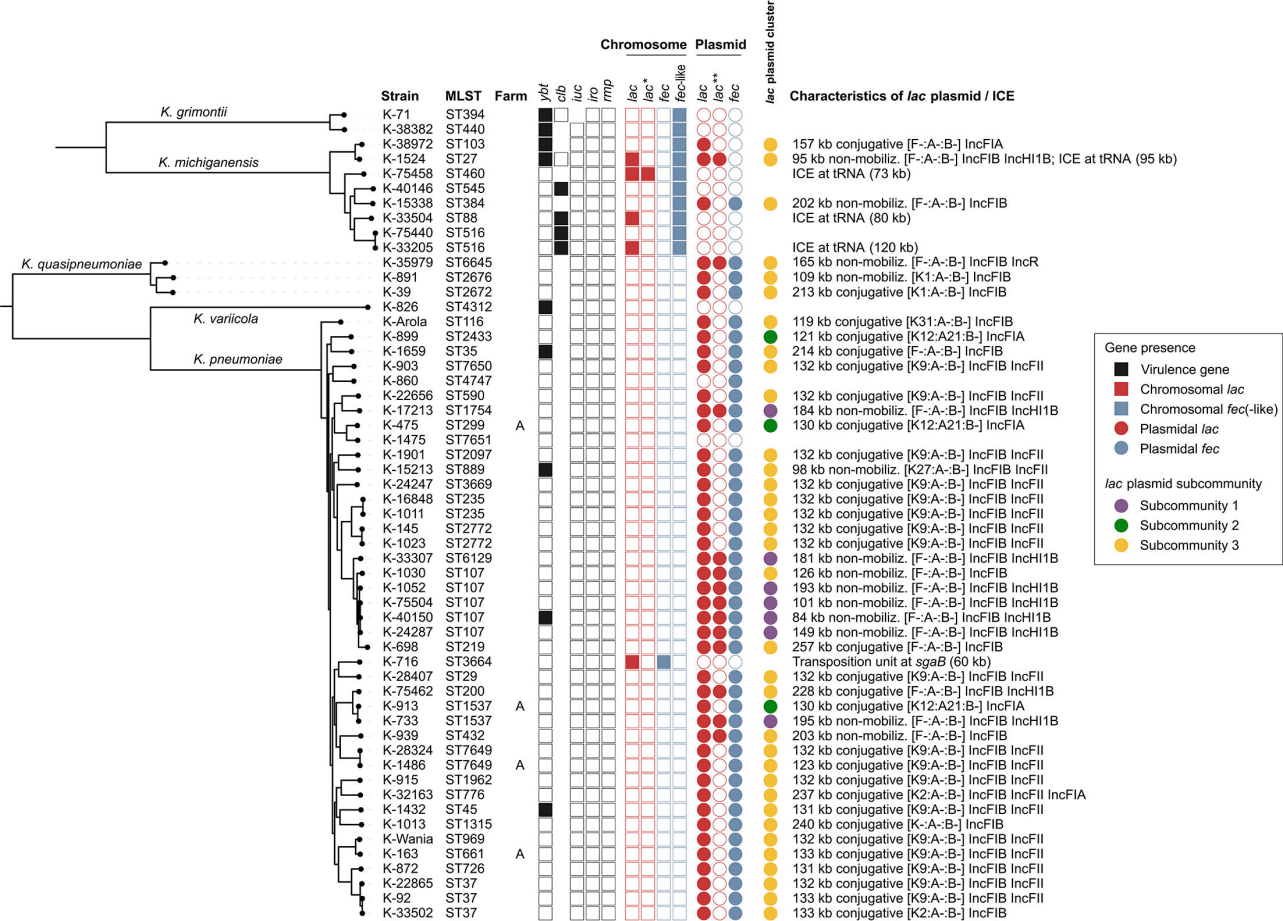
Population structure and characteristics of 55 unique *Klebsiella* spp. isolates associated with mastitis. The presence and location of acquired *lac*_acq_, *fec*, and important virulence genes are indicated. Shared *lac*_acq_-carrying mobile genetic elements are colored according to their pling subcommunity in green, yellow, or purple. Characteristics of *lac*_acq_,-carrying plasmids (replicon types, pMLST, and plasmid sizes) and ICE (integration site, size) are described. Phylogenies are based on 169,256 and 300,953 parsimony-informative sites from core genome alignments of 2.95 Mb (*K. pneumoniae* complex) and 4.86 Mb (*Klebsiella oxytoca* complex), respectively. *Second chromosomal acquired *lac*_acq_ copy; **second plasmidal acquired *lac*_acq_ copy; ICE, integrative conjugative element.

Overall, the 60 mastitis isolates were phylogenetically diverse and belonged to 44 sequence types (Fig. 1). Among the 41 deduplicated (unique) *K. pneumoniae* strains (i.e., pairwise cgMLST distance >10 alleles), ST107 was the most frequent sequence type (5/41, 12.2%), observed across five different farms, followed by ST37 (3/41, 7.3%) (Fig. 1). Two additional isolates were closely related to ST107 and assigned to ST219 and ST6119, both differing at one MLST locus from ST107. Phylogenetic analysis with public ST107 genomes showed that Swiss ST107 mastitis isolates clustered in clades dominated by *lac*_acq_^+^ *fec*^+^ bovine isolates and distinct from human-associated lineages, which largely lacked *lac*_acq_ and *fec* (Fig. S1). Overall, 61/61 of the bovine ST107 isolates carried *lac*_acq_ and *fec*, versus 23/135 (*lac*_acq_) and 22/135 (*fec*) of the human ST107 isolates (Fisher’s exact test, *P* < 0.001) (Table S2).

### Distribution of virulence determinants

Of the major *Klebsiella* virulence factors *ybt*, *clb*, *iro*, *iuc*, and *rmpA/rmpA2*, only *ybt* (yersinibactin) and *clb* (colibactin) were sporadically detected: *ybt* was present in 4 (9.8%) of the 41 unique *K. pneumoniae*, 2/8 (25.0%) *K*. *michiganensis*, and 2/2 (100%) *K*. *grimontii* isolates (Fig. 1). The *clb* gene was found in 4/8 (50%) *K*. *michiganensis* isolates (Fig. 1).

Previous studies have suggested that a combination of acquired *lac*_acq_ and *fec* operons likely provides bovine mastitis *K. pneumoniae* with the ability to invade and thrive within mammary epithelial cells (12, 15). Here, the acquired *lac*_acq_ operon was detected in 48/55 (87.3 %) isolates: in 39/41 (95.1 %) *K*. *pneumoniae*, 6/8 (75 %) *K*. *michiganensis*, and 3/3 (100 %) *K. quasipneumoniae*, but not in *K. grimontii* (0/2) and *K. variicola* (0/1) (Fig. 1). The *fec* gene cluster was detected in 53/55 (96.4 %) isolates: in all isolates except one *K. pneumoniae* (K-1475) and the *K. variicola* (K-826) isolate.

### Mobile genetic elements harboring *lac*_acq_ and *fec*

To better understand the genetic context and the mechanisms responsible for the transfer of *lac*_acq_ and *fec*, we resolved the chromosomes and plasmids of the 55 deduplicated strains collected during this study.

Among the 42 *lac*_acq_-positive *K. pneumoniae* and *K. quasipneumoniae* isolates, *lac*_acq_ was almost exclusively (41/42, 97.6%) detected on plasmids, all of which co-harbored the *fec* gene cluster. Twelve out of the 41 plasmids (28.6%) contained two *lac*_acq_ copies. The plasmids ranged in size from 84 to 257 kb and carried IncFIB (*n* = 38) or IncFIA (*n* = 4) replicons, often in combination with other replicon types (Fig. 1). Besides *lac*_acq_ and *fec*, the plasmids often contained heavy metal resistance genes, such as the arsenate resistance operon *ars* (17/41, 41.5%), the Copper Homeostasis and Silver Resistance Island (CHASRI) with the *sil* and *pco* operons (11/41, 26.8%), or the tellurite resistance operons *ter* (5/41, 12.2%). Antimicrobial resistance genes were detected in 8/41 (19.5%) plasmids (Table 1). Exceptionally, isolate *K. pneumoniae* K-716 carried *lac*_acq_ and *fec* on a 60 kb chromosomal unit flanked by transposases. Querying the NCBI core nucleotide database revealed an identical (>99% coverage and identity) region on *K. pneumoniae* plasmid CP092409.1 (Fig. S2).

**TABLE 1:** Acquired antimicrobial resistance genes and their genetic context in mastitis-associated *Klebsiella* spp.^*a*^

Isolate	Species	Sequence type	Acquired antimicrobial resistance genes	Location of resistance genes	Plasmidal co-location of *lac*_acq_ and *fec*
K-1013	*K.p*.	ST1315	*aadA2, cmlA1, aadA1, aac(3)-IId, blaTEM-1, dfrA12, floR, mef(B), qnrS1, tet(A), sul2, sul3*	Plasmidal: IncFIB(K) [F-:A-:B-]	*lac*_acq_ *^+^, fec^+^*
K-1659	*K.p*.	ST35	*blaSHV-1, tet(D*)	Plasmidal: IncFIB(K) [F-:A-:B-]	*lac*_acq_ *^+^, fec^+^*
K-1052	*K.p*.	ST107	*aph(3'')-Ib, aph(6)-Id*	Plasmidal: IncFIB(K), IncHI1B [F-:A-:B-]	*lac*_acq_*^+^, lac*_acq_ *^+^, fec^+^*
K-17213	*K.p*.	ST1754	*aph(3'')-Ib, aph(6)-Id, tet(B*)	Plasmidal: IncFIB(K), IncHI1B [F-:A-:B-]	*lac*_acq_ *^+^, lac*_acq_ *^+^, fec^+^*
K-33307	*K.p*.	ST6129	*aph(3'')-Ib, aph(6)-Id*	Plasmidal: IncFIB(K), IncHI1B [F-:A-:B-]	*lac*_acq_ *^+^, lac*_acq_ *^+^, fec^+^*
K-733	*K.p*.	ST1537	*aph(3'')-Ib, aph(6)-Id*	Plasmidal: IncFIB(K), IncHI1B [F-:A-:B-]	*lac*_acq_ *^+^, lac*_acq_ *^+^, fec^+^*
K-698	*K.p*.	ST219	*aph(3'')-Ib, aph(6)-Id*	Plasmidal: IncFIB [F-:A-:B-]	*lac*_acq_ *^+^, lac*_acq_ *^+^, fec^+^*
K-75462	*K.p*.	ST200	*aph(3'')-Ib, aph(6)-Id*	Plasmidal: IncFIB(K), IncHI1B [F-:A-:B-]	*lac*_acq_ *^+^, lac*_acq_ *^+^, fec^+^*

^*a*^ K.p., *Klebsiella pneumoniae*.

**Fig 2:**
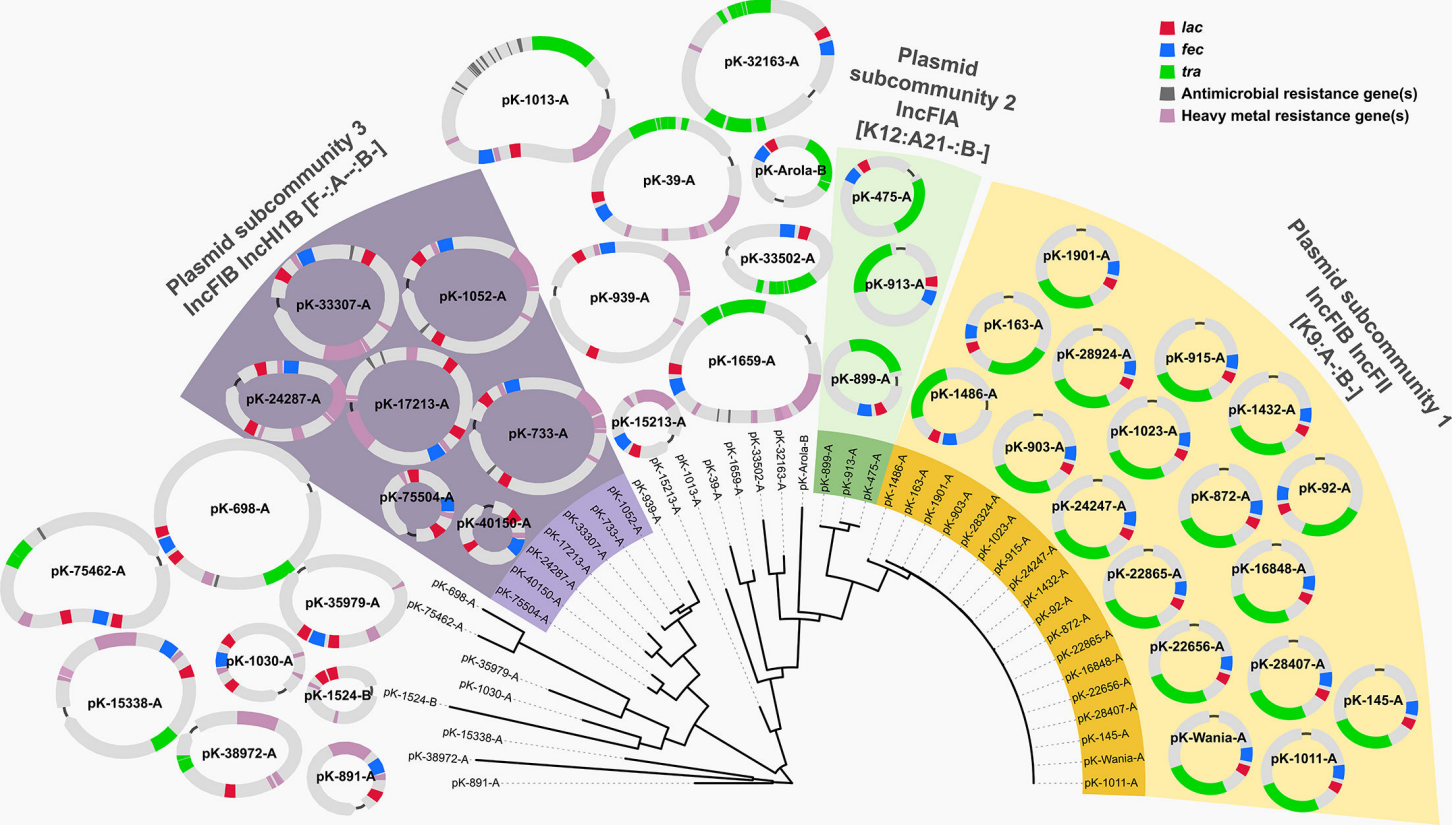
Diversity of *lac*_acq_-carrying plasmids from 44 mastitis-associated *Klebsiella* spp. isolates. Closely related plasmids forming subcommunities (DCJ-Indel distance ≤ 5, containment distance < 0.5) are shaded in purple, green, or yellow. The tree was constructed using the neighbor-joining algorithm, based on pairwise Mash distances between plasmids using Mashtree 1.2.0 (17). Plasmid assembly graphs were visualized with Bandage (18). The presence of *lac*_acq_, *fec*, *tra*, antimicrobial resistance genes, and heavy metal resistance genes is labeled.

**Fig 3:**
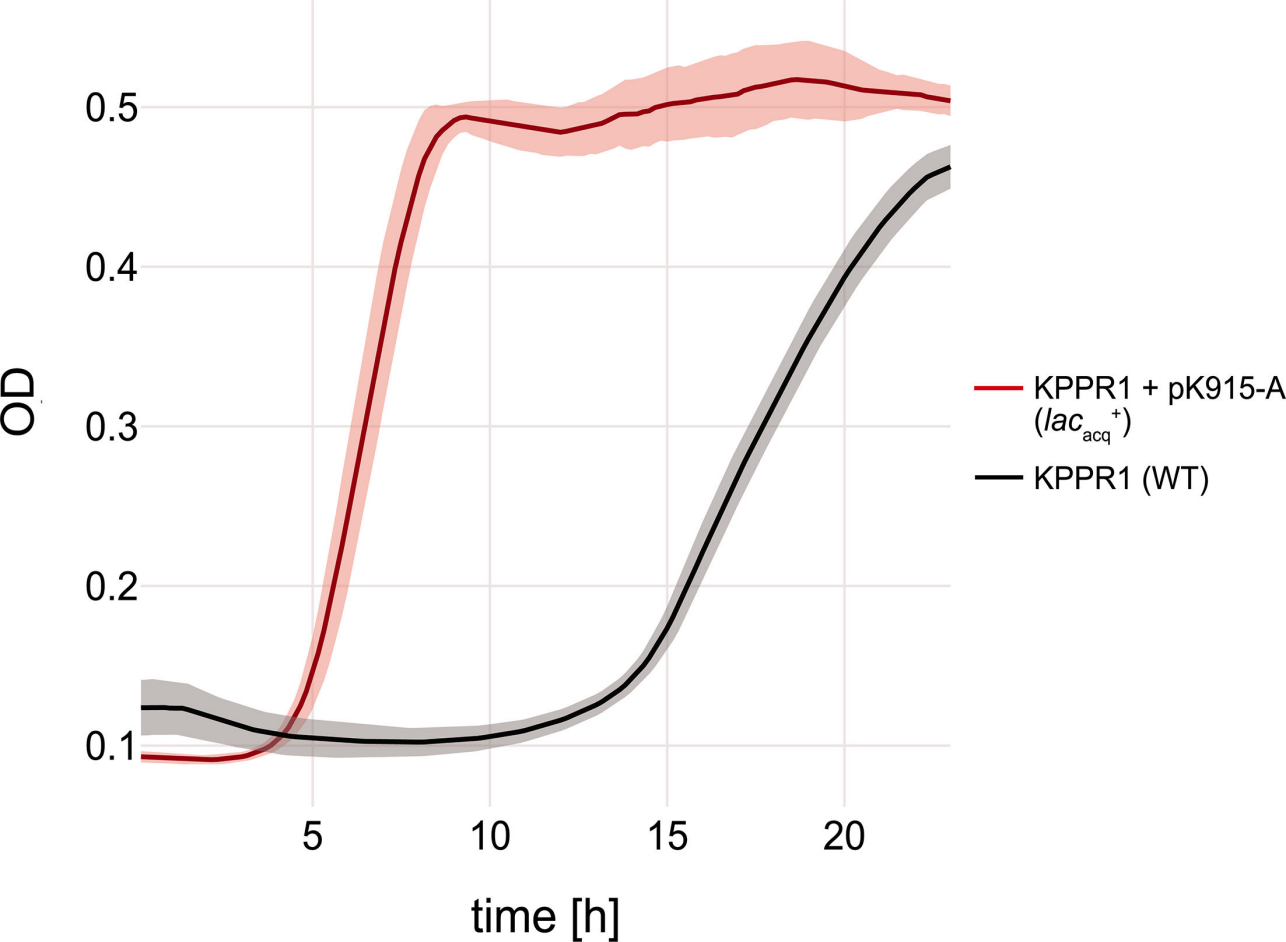
Growth advantage conferred by *lac*_acq_^+^ plasmids in lactose minimal media. Growth curves of wild-type *K. pneumoniae* KPPR1 (black) and the transconjugant KPPR1 + pK-915-A [*lac*_acq_^+^] (red) in M9 minimal medium supplemented with lactose as the sole carbon source. Lines represent the mean, and shaded areas indicate the standard deviation of replicates.

Unexpectedly, 18 *K*. *pneumoniae* isolates belonging to 14 sequence types shared a near-identical (>99.9% identity), conjugative IncFIB(K) IncFII(pKP91)[K9:A-:B-] plasmid containing *lac*_acq_ and *fec* (Fig. 1 and 2). Eleven of these 18 plasmids were identical in size (131,579 bp) and differed at only a few (0 to 7) nucleotide positions, suggesting recent sharing and high conjugation potential. The remaining seven plasmids of this subcommunity had minor structural variations, resulting in plasmid sizes of 123 to 133 kb (Fig. S3). Besides *lac*_acq_ and *fec*, the plasmids largely consisted of transfer (*tra*) genes facilitating conjugation and did not contain resistance or additional virulence genes. Three genetically unrelated isolates (ST299, ST661, and ST1537) that harbored this plasmid originated from the same farm (farm A), suggesting that a plasmid outbreak contributed to the high case numbers at farm A, in addition to a clonal outbreak. The plasmid had no identical matches in the NCBI Core Nucleotide database but shared its backbone (>95% sequence identity over up to 74% of the query length) with known sequences. Conjugation assays confirmed that the plasmid transfers efficiently, with a frequency of 5.25 × 10⁻³ transconjugants per donor cell. In minimal media supplemented with lactose as the sole carbon source, the transconjugant strain (KPPR1 + pK-915-A [*lac*_acq_^+^]) showed a substantially reduced lag phase (3.2 ± 0.3 h vs 11.4 ± 1.0 h [mean ± SD]) and faster doubling times (0.5 ± 0.1 h vs 1.6 ± 0.2 h [mean ± SD]) compared to the wild-type KPPR1 which lacks *lac*_acq_ (Fig. 3). Growth in glucose minimal medium did not differ between the strains.

Rearrangement and containment analyses with pling (19) identified two additional plasmid subcommunities consisting of (i) three IncIA(HI1) [K12:A21:B-] conjugative plasmids (subcommunity 2) and (ii) seven non-mobilizable IncFIB(K)(pCAV1099-114) IncHI1B(pNDM-MAR) [F-:A-:B-] plasmids (subcommunity 3), respectively (Fig. 1 and 2). Plasmids of subcommunity 3 were primarily detected in the ST107 lineage. They contained two *lac*_acq_ copies and, occasionally, heavy metal and aminoglycoside resistance genes (*aph(3'')-Ib* and *aph(6)-Id*).

**Fig 4:**
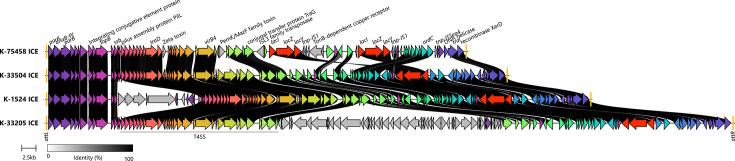
Comparison of *lac*_acq_-carrying ICE in *K. michiganensis* chromosomes. Gray shading between homologous genes indicates sequence identity above 70%, with darker shades representing higher percent identity as shown in the legend. Left and right ICE attachment sites (*attL* and *attR*) are labeled in orange.

Among the six *lac*_acq_^+^ *K. michiganensis* isolates, the *lac*_acq_ operon was detected either on integrative conjugative elements (ICEs; *n* = 4) or on plasmids (*n* = 3). The three plasmids were unrelated and carried *fec* in combination with one or two *lac*_acq_ copies (Fig. 1). The ICEs were always integrated at the tRNA^phe^ locus and harbored one (*n* = 3) or two (*n* = 1) *lac*_acq_ copies. Although the isolates were phylogenetically distant, three ICE (in K-33504, K-1524, K-33205) were highly homologous (average nucleotide identity [ANI] > 99.5%). The ICE sequence of K-33504 was fully contained within those of K-1524 and K-33205; the latter additionally harbored a prophage, while K-1524 carried an additional transposable element (Fig. 4). The fourth ICE (K-75458) shared a lower sequence identity (ANI 96%) with the others and showed additional rearrangements, including a second *lac*_acq_ copy.

Irrespective of horizontal *fec* acquisition, all *K. michiganensis* and *K. grimontii* isolates harbored a *fec*-like operon in the chromosome. These chromosomal *fec*-like operons differed from the plasmidal *fec*, with pairwise sequence identities of the individual genes (*fecABCDE*) ranging between 66% and 88%. The absence of mobility-associated genes in the surrounding region suggests that these *fec*-like genes are part of their native genome.

Acquired antimicrobial resistance genes were uncommon among the mastitis isolates and predominantly comprised the aminoglycoside resistance genes *aph(3'')-Ib* and *aph(6)-Id* (Table 1). Only two isolates carried acquired beta-lactam resistance genes (*bla*_TEM_ or *bla*_SHV_). All resistance genes were detected in *lac*_acq_^+^ and *fec*^+^ *K. pneumoniae* plasmids. Notably, one plasmid (pK-1013-A) carried a class 1 integron with a *dfrA12–aadA2* cassette array, forming part of a class 1 integron-associated complex resistance locus (CRL) that also harbored additional AMR genes in its vicinity.

## DISCUSSION

In this study, we combined short-read and long-read sequencing to investigate the genomes of 60 *Klebsiella* isolates associated with bovine mastitis. The high prevalence (>95%) of *lac*_acq_ and *fec* in *K. pneumoniae* is consistent with previous studies in the USA and China, where these key determinants of bovine mastitis were detected in 85%–99% of the isolates (12, 14, 20). We demonstrate that this association also extends to other *Klebsiella* species: 75% of the *K. michiganensis* mastitis isolates harbored an acquired *lac*_acq_*,* while a *fec*-like gene cluster appeared to be intrinsic to *K. michiganensis* and the closely related *K. grimontii*. Similarly, Blum et al. found that the *fec* system is enriched among bovine mastitis-associated *Escherichia coli* (21). Their functional analyses showed that *fec* expression is strongly induced in milk, promotes bacterial growth in this niche, and is essential for the development of mastitis in cows. Given the close relationship between *E. coli* and *Klebsiella*, it is plausible that *fec* fulfills a comparable role in mastitis pathogenesis. In addition, our growth experiments demonstrate that acquisition of *lac*_acq_ confers a strong fitness benefit under lactose limitation. Although *Klebsiella* spp. carry an intrinsic chromosomal *lac* operon, *lac*_acq_^+^ transconjugant strains exhibited substantially shorter lag times and increased growth rates. This indicates that the plasmid-encoded lactose metabolism is not redundant, but instead provides enhanced capacity, faster induction, deregulated expression, or more efficient lactose uptake compared to the chromosomal system.

Our analysis of mobile genetic elements revealed that the dissemination of co-located *lac*_acq_ and *fec* is largely driven by plasmids, with ICE contributing to the spread of *lac*_acq_ in *K. michiganensis*. Most *lac*_acq_^+^ plasmids carried an IncFIB family replicon, which is the dominant replicon type of resistance and virulence plasmids in *K. pneumoniae* (22). Notably, a single ~132 kb conjugative *lac*_acq_^+^ *fec*^+^ plasmid was detected in nearly half of the *K. pneumoniae* isolates, despite their phylogenetic diversity and origin from geographically distant farms in Switzerland. This IncFIB(K)/IncFII(pKP91) [K9:A-:B-] plasmid likely represents a highly transmissible and structurally stable plasmid that has recently disseminated and confers a strong selective advantage. This is remarkable given that *K. pneumoniae* plasmids are typically highly dynamic with extensive recombination and rearrangements (23, 24). A similar exception is the *bla*_OXA-48_-carrying IncL/M plasmid, an important cause of carbapenem resistance, which is highly conserved across lineages (23, 25). Other examples of horizontally spreading but structurally stable plasmids in Enterobacterales include the epidemic *mcr-1*-carrying IncI2, IncHI2, and IncX4 plasmids in *E. coli* from swine and poultry (26) and *bla*SHV-12-carrying IncX3 plasmids circulating in poultry farms (27). Besides antimicrobial selection, additional factors, such as close animal proximity, manure reuse as fertilizer, farm-to-farm networks, and co-selection by heavy metals, may contribute to the persistence and dissemination of these plasmids. Previous studies have shown that genes conferring resistance to copper, silver, and arsenic are more prevalent in mastitis-associated strains than in isolates from other sources (14, 28). Consistently, in our isolate collection, *lac*_acq_^+^ *fec*^+^ plasmids frequently co-harbored heavy metal resistance. In dairy cows, heavy metals are generally provided only in trace amounts through mineral supplements. This differs from pigs and poultry, where higher doses of heavy metals are sometimes used for growth promotion or disease control, creating co-selection pressure that can favor the spread of antimicrobial resistance genes (29).

Although mastitis is the most common indication for antimicrobial use in Swiss dairy cows (30), acquired resistance genes were only sporadically detected among the mastitis isolates. The relatively low rate of resistance may reflect the EU-wide ban on antimicrobial growth promoters enforced in 2006 (EC no. 1831/2003), which also applies to Switzerland, and the tightly regulated use of antimicrobials for both food and non-food-producing animals in Switzerland (30, 31). Nevertheless, the co-occurrence of *lac*_acq_, *fec*, heavy metal, and antimicrobial resistance genes on multiple plasmids highlights the potential for co-selection within the dairy environment.

In agreement with earlier studies (12, 14), the *K. pneumoniae* mastitis isolates belonged to diverse lineages. Most of the 31 STs were represented by only one isolate. Despite this diversity, ST107 was markedly more common (5/41, 12%). This sequence type has previously been identified as a globally prevalent mastitis-associated clone and was dominant in isolate collections from Canada (32), China (20), Scotland (1), and the USA (14, 33). Unlike most other mastitis isolates, the analyzed ST107 isolates harbored non-mobilizable plasmids encoding two *lac*_acq_ operons. The conserved architecture of these plasmids suggests stable carriage and vertical inheritance within ST107, likely contributing to the global success of this lineage as a bovine pathogen. ST107 is also known as a human pathogen: a recent study identified ST107 as a dominant cause of human bloodstream infections in Norway (34). However, these clinical isolates rarely carried *lac*_acq_ or *fec* (Fig. S1), suggesting that different ST107 sublineages may circulate in distinct ecological or clinical contexts.

Other *K. pneumoniae* STs affecting multiple cows within and across herds included ST661, ST299, and ST432, each encompassing a cgMLST transmission cluster. ST661 is a rare lineage that is associated with multidrug resistance in clinical settings and has been implicated in nosocomial infections and hospital outbreaks in Italy and the UK (35, 36). Likewise, ST37, isolated from three different milk samples in this study, is a clinically important lineage associated with the dissemination of extended-spectrum ß-lactamase and carbapenemase resistance genes (37, 38). The presence of *Klebsiella* STs associated with human disease emphasizes a potential public health risk posed by bovine mastitis. A recent large-scale One Health study found *K. pneumoniae* populations from human and animal sources to be distinct but overlapping, with transmission occurring mainly between humans but occasional spillover from animals (39). Comparative genomics further indicates that bovine and human isolates share high overall genomic similarity and did not identify obvious genetic barriers to cross-host transmission, although key virulence factors associated with *K. pneumoniae* infections in humans were largely absent in strains linked to bovine mastitis (12, 15). Accordingly, genes encoding yersiniabactin, salmochelin, aerobactin, colibactin, the hypermucoidy locus *rmpADC*, and the alternative hypermucoidy marker gene *rmpA2* were, for the most part, not detected in our study. Potential transmission routes to humans include direct contact during milking, exposure to contaminated equipment, raw milk consumption, or environmental dissemination via manure.

This study has notable limitations. As isolates originated from diagnostic submissions, our data set may over-represent acute mastitis cases that were more severe or unresponsive to initial treatment, rather than capturing the full diversity of *Klebsiella*-associated mastitis. Furthermore, most farms contributed only a single isolate, limiting our ability to assess persistence, within-farm diversity, or relative abundance of specific clonal types. Future studies should include environmental sampling of abiotic farm surfaces to better understand potential reservoirs and transmission pathways.

In conclusion, our study demonstrates that plasmids are the primary vehicles of the mastitis-associated determinants *lac*_acq_ and *fec* in *Klebsiella* spp. Both horizontal and vertical transmission contribute to their spread, with a single, highly transmissible plasmid circulating among phylogenetically diverse strains across Swiss farms significantly driving the burden of mastitis. Our findings emphasize the importance of monitoring mobile genetic elements in veterinary pathogens, as they can facilitate rapid adaptation and persistence in agricultural settings.

## MATERIALS AND METHODS

### Bacterial isolates

*Klebsiella* strains were isolated from individual quarter milk samples from cattle with mastitis. Diagnosis was performed by the veterinarian in charge according to a standardized procedure. A total of 36 strains were obtained from the ambulatory veterinary hospital of the University of Zurich, which serves the canton of Zurich and the surrounding region, and from the clinic of reproductive medicine of the veterinary hospital of the University of Zurich. Milk samples were submitted to the routine mastitis diagnostic laboratory of the Institute for Food Safety and Hygiene in Zürich, and bacteria were isolated following the National Mastitis Council guidelines (40).

Another 24 *Klebsiella* isolates were forwarded to the Institute for Food Safety and Hygiene by a veterinary diagnostic laboratory that provides disease diagnostic services to veterinarians and their clients in Switzerland.

All 60 *Klebsiella* isolates were collected from individual cows from 49 different dairy farms located throughout Switzerland between December 2022 and August 2024.

Species identification was confirmed by matrix-assisted laser desorption/ionization time of flight (MALDI-TOF) spectrometry (Bruker Daltonics) using the software Flex Control 3.4, the MALDI Biotyper (MBT) Compass database version 4.1.100.10, and the MBT Compass BDAL Library March 2023, according to the manufacturer’s instructions. Isolates were cultured on sheep blood agar plates (24 h at 37°C), transferred into a glycerol freezing medium, and stored at −80°C until further processing.

### DNA extraction and whole genome sequencing

DNA was extracted from single purified colonies grown on Columbia sheep blood agar (Difco) using the MagPurix Bacterial DNA Extraction Kit (Zinexts). For short read sequencing, libraries were prepared with the Nextera DNA Flex Library Preparation Kit (Illumina) and sequenced using the Illumina MiniSeq platform with 2 × 150 bp paired-end chemistry.

For long-read sequencing, libraries were prepared using the SQK-RBK114.24 Rapid Barcoding Kit (Oxford Nanopore) according to the “Nanopore-only Microbial Isolate Sequencing Solution” protocol. Libraries were sequenced on MinION devices using R10.4.1 flow cells.

### Genomic analyses

Illumina read adapters and low-quality bases were trimmed with fastp 0.22.0 (41). Dorado 0.7.0 (github.com/nanoporetech/dorado) with the sup@v5.0 (dna_r10.4.1_e8.2_400bps_sup@v5.0.0) model was used for simplex basecalling, demultiplexing, and adapter and barcode trimming of Nanopore data. Nanopore reads were filtered to a minimum read length of 1,000 bp and quality controlled using nanoq 0.10.0 (42). Hybrid assemblies were generated using Unicycler v0.5.0 (43) with default settings. Assembly quality was assessed using QUAST 5.0.2 (44) and CheckM v1.1.3 (45). Kleborate v2.0.4 (46) was used for taxonomic assignment and virulence gene detection. Sequence types were identified with mlst 2.22.0 (github.com/tseemann/mlst), which relies on PubMLST (47). cgMLST distances were determined using pyMLST 2.1.6 (options -c 0.9 -i 0.9) (48) in combination with the *K. pneumoniae* species complex (2,358 loci) or *K. oxytoca* species complex schemes (2,536 loci) (49) available at cgmlst.org. Putative transmission clusters were confirmed by core genome SNP (cgSNP) analysis using the CFSAN SNP Pipeline v2.2.1 (50) (default settings) with Illumina read data, run separately for each sequence type with a corresponding hybrid assembly as reference.

Core genome alignments were generated for each species complex separately with parsnp 1.5.3 (51) and used to construct maximum-likelihood phylogenetic trees using IQ-TREE 2.0.3 (52), applying the generalized time-reversible model with gamma distribution. Phylogenetic trees were visualized in iTOL v6 (53). The *lac*_acq_ and *fec* operons were identified using ABRicate 1.0.0 (https://github.com/tseemann/abricate) (minimum sequence coverage/identity 70%/90%) with a custom database containing the *lac*_acq_ and *fec* sequences extracted from the annotated assembly of plasmid pK-Wania-A (CP184439). Antimicrobial and heavy metal resistance genes were identified using AMRFinderPlus 3.10.24 (54). Plasmid replicon types were identified with ABRicate v1.0.0 in conjunction with the PlasmidFinder database (minimum sequence coverage/identity 60%/95%) (55). pMLST types were identified using PubMLST.org (47, 55). Clusters of related plasmids (subcommunities) were determined using pling v2.0.0 (19) with a containment distance threshold of 0.5 and a Double Cut and Join Indel distance threshold of 5. Plasmid mobility was predicted using MOB-typer v3.1.7 (56). SNPs between K9:A-:B- plasmids were determined by mapping reads to the plasmid pK-Wania-A (CP184439) as reference using breseq 0.39.0 (57). Putative integrative conjugative elements and their attachment sites were detected using ICEfinder (accessed 04/12/2024) in combination with the ICEberg3 database (58). Structural comparisons of mobile genetic elements were generated and visualized with Clinker v0.0.31 (59) or Easyfig 2.2.5 (60).

### Plasmid conjugation experiments

Conjugation experiments were performed with the plasmid-free, rifampicin-resistant recipient strain *K. pneumoniae* KPPR1 (61). Unlike *lac*_acq_^+^ strains, KPPR1 displays strongly reduced growth on M9 minimal agar plates with lactose, where it forms only small colonies. Single colonies of the donor (strain K-915) and recipient were inoculated in LB broth and grown overnight at 37°C. Subsequently, equal volumes of the donor and recipient cultures were mixed and incubated overnight at 37°C without shaking. Serial dilutions of the mixed culture were plated on selective plates on which the donor strain and the transconjugant can grow (M9 minimal media with 0.2% lactose), as well as on plates on which only the transconjugants can grow (M9 minimal media with 0.2% lactose and 100 µg/mL rifampicin). The transfer frequency was calculated as the quotient of the number of transconjugants over the number of transconjugants plus donors.

### Growth curve assays

Bacterial growth was assessed in 96-well microtiter plates using M9 minimal medium supplemented with either 0.2% lactose or 0.2% glucose as the sole carbon source. Strains were grown overnight in LB media at 37°C, washed twice in M9 without a carbon source, and resuspended to OD₆₀₀ =0.1. Cultures were inoculated into microplates at a starting OD₆₀₀ of 0.01 (20 µL inoculum into 180 µL medium) in biological triplicates. Plates were sealed, incubated at 37°C with orbital shaking, and OD₆₀₀ was recorded every 10 min for 24 h using a microplate reader (Tecan Spark). Growth parameters (lag time, doubling time) and growth curve visualizations were obtained with Dashing Growth Curves (62).

## Data Availability

The data sets generated during this study are available in the NCBI repository under BioProject accession number PRJNA1224596. Individual accession numbers are listed in Table S1.

## SUPPLEMENTAL MATERIAL

Supplemental figures aem.01162-25-s0001.pdf Figures S1 to S3.

Supplemental tables aem.01162-25-s0002.xlsx Tables S1 and S2.

ASM does not own the copyrights to Supplemental Material that may be linked to, or accessed through, an article. The authors have granted ASM a non-exclusive, world-wide license to publish the Supplemental Material files. Please contact the corresponding author directly for reuse.
