# Supplementary material for: Genomic characterization of Klebsiella spp. from bovine mastitis: dissemination of a conserved, highly transmissible lacacq+ fec+ plasmid drives burden of disease
Source: Appl Environ Microbiol. 2025 Nov 20;91(12):e01162-25. doi: 10.1128/aem.01162-25 (PMC12724306; doi:10.1128/aem.01162-25)
Supplement: Supplemental figures — Figures S1 to S3. [file aem.01162-25-s0001.pdf]

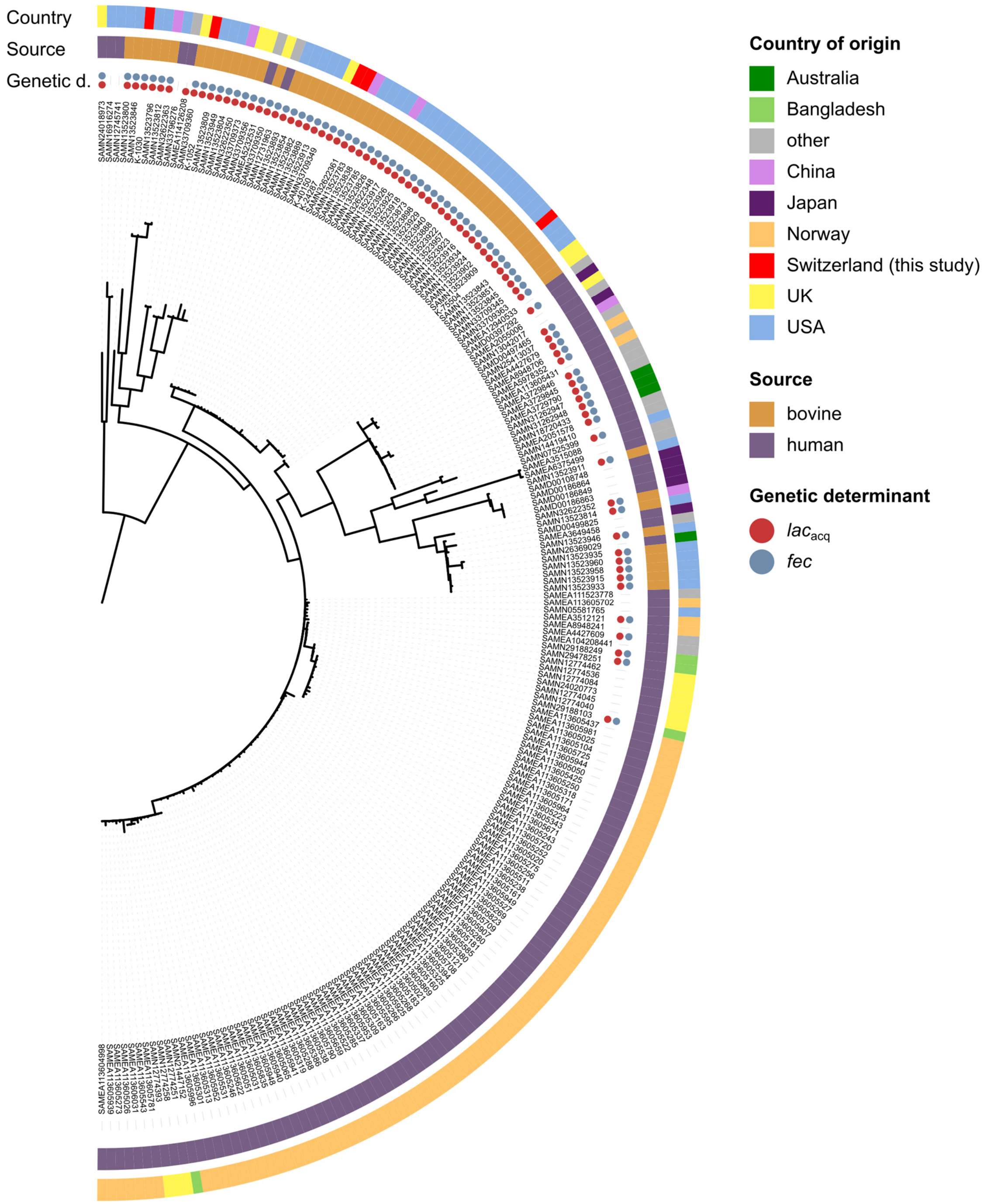

Supplementary Figure 1. Phylogenetic tree of five Swiss ST107 mastitis isolates in the context of 191 global ST107 isolates of bovine or human origin. The country of origin, host, and presence of lac_{acq} and *fec* are labelled according to the legend. Assemblies of global isolates were retrieved from pathogenwatch (accessed 01/06/2025). Of the 135 human isolates, 85 originate from human bloodstream infections in Norway. The phylogeny is based on 7,209 parsimony-informative sites identified in a 694,141 bp core genome alignment.

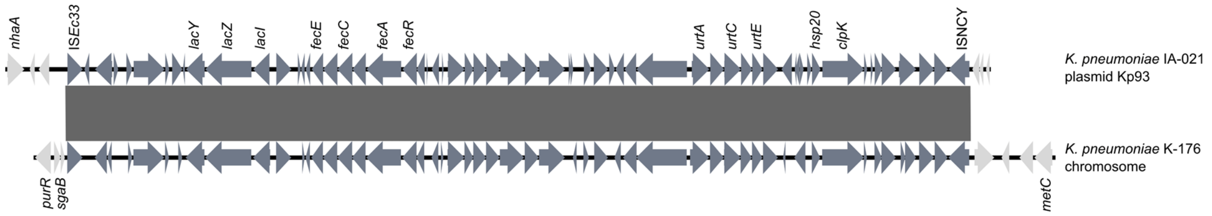

Supplementary Figure 2. Alignment of the *lac*$_{acq}$-containing regions in the chromosome of *K. pneumoniae* K-176 and the *K. pneumoniae* plasmid Kp93 (CP092409.1). Grey shading between sequences indicates regions with >99% sequence identity.

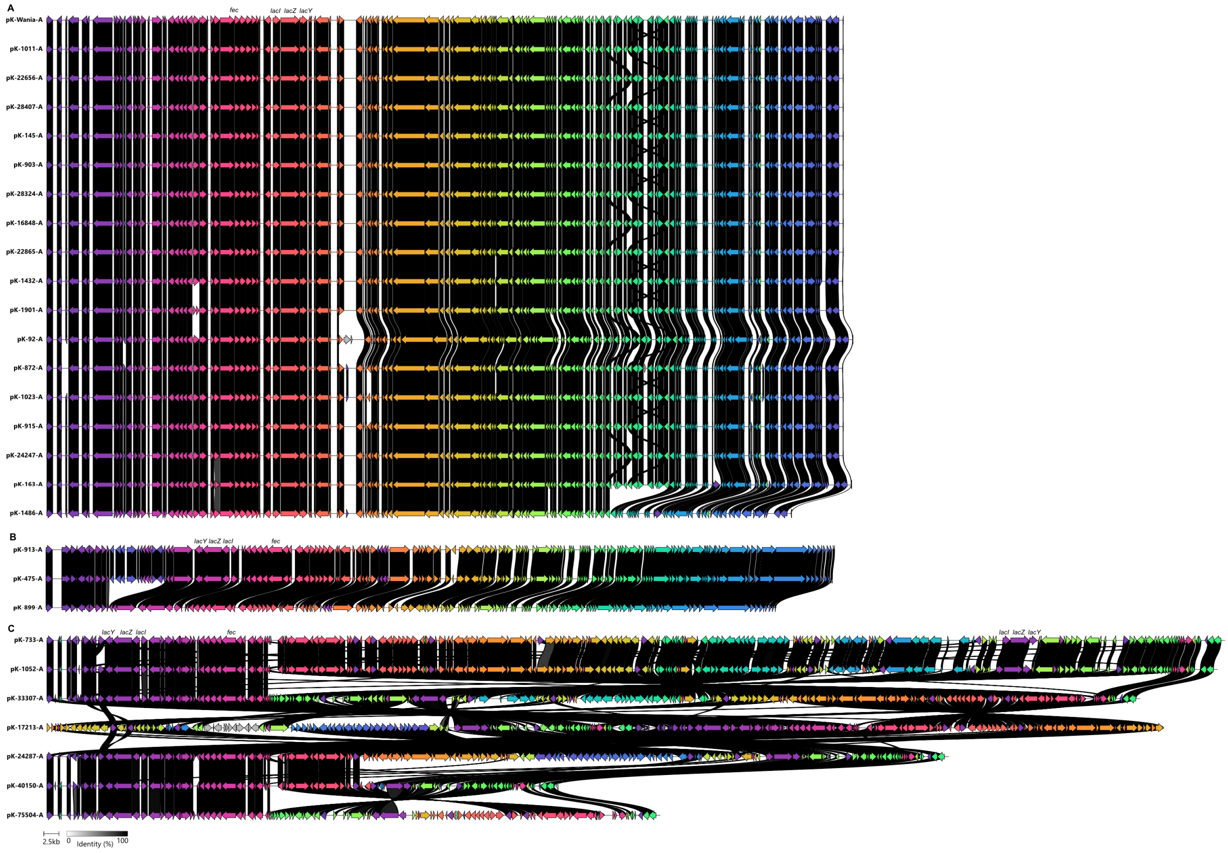

Supplementary Figure 3. Alignment of *lac*$_{acq}$-containing *K. pneumoniae* plasmids from the plasmid subcommunities 1 (A), 2 (B) and 3 (C). Grey shading between homologous genes indicates sequence identity above 70%, with darker shades representing higher percent identity as shown in the legend.
